# Association of visceral adiposity index with asymptomatic intracranial arterial stenosis: a population-based study in Shandong, China

**DOI:** 10.1186/s12944-023-01831-1

**Published:** 2023-05-17

**Authors:** Weihua Zhao, Xiaotong Ma, Jiachen Ju, Yuanyuan Zhao, Xiang Wang, Shan Li, Yanling Sui, Qinjian Sun

**Affiliations:** 1 Department of Neurology, Shandong Provincial Hospital, Shandong University, Jinan, Shandong China; 2 Department of Neurology, Shandong Provincial Hospital Affiliated to Shandong First Medical University, No. 324 Jingwu Weiqi Road, Jinan, Shandong 250021 P.R. China

**Keywords:** Asymptomatic intracranial atherosclerosis, Obesity indictor, Visceral adiposity index

## Abstract

**Background and objective:**

The visceral adiposity index (VAI), as a composite indictor to evaluate visceral adipose function, has been demonstrated to be correlated with atherosclerosis. The study objective was to explore the association between asymptomatic intracranial arterial stenosis (aICAS) and VAI in Chinese rural dwellers.

**Methods:**

The cross-sectional study consisted of 1942 participants  ≥ 40 years old who were living in Pingyin County, Shandong Province and free from history of clinical stroke and transient ischemic attack. The aICAS in the study was diagnosed by transcranial doppler ultrasound combined with magnetic resonance angiography. The multivariate logistic regression models were deployed to explore the correlation of VAI with aICAS, and receiver operating characteristic (ROC) curve were plotted to compare the performance of models.

**Results:**

The participants with aICAS comparing to those without had a significantly higher VAI. After adjusting for confounding factors including age, hypertension, DM, sex, drinking habit, LDL-C, hsCRP, and smoking habit, the VAI-Tertile 3 (vs. VAI-Tertile 1) was positively associated with aICAS (OR, 2.15; 95% CI, 1.25–3.65; *P* = 0.005). The VAI-Tertile 3 was still markedly associated with aICAS among the underweight and normal weight (BMI ≤ 23.9 kg/m^2^) participants (OR, 3.17; 95% CI, 1.15–8.71; *P* = 0.026) with an AUC = 0.684. A similar relationship between VAI and aICAS was obtained among the participants with no abdominal obesity (WHR < 1, OR, 2.03; 95% CI, 1.14–3.62; *P* = 0.017).

**Conclusions:**

The possible correlation between VAI and aICAS was found to be positive for the first time among Chinese rural residents over 40 years old. A higher VAI was found to be significantly associated with aICAS among the participants who were underweight or normal weight, and these results may provide additional risk stratification information for aICAS.

## Introduction

Stroke has already become the second-leading cause of death, which poses considerable challenges to the world [1] especially in lower-middle income countries [2, 3]. Intracranial arterial stenosis (ICAS) is currently considered to be one of the most prevalent causes of stroke and one of the most important risks of recurrent stroke, particularly in Asians [4–7]. Intracranial arterial stenosis can remain asymptomatic for a long time before stroke occurs. Asymptomatic ICAS (aICAS) is the pre-disease stage of ICAS and magnifies the global burden of ICAS; meanwhile, it is increasingly considered a contributing risk for ischemic stroke. Early recognition and management of aICAS is of great importance in the primary prevention of stroke.

Obesity has already been regarded as a possible contributor to aICAS. It has aroused widespread concern with increasing prevalence and disease burden [8, 9]. Obesity is classified as central obesity and peripheral obesity. Central obesity, also called abdominal obesity, can be further divided into subcutaneous fat-based abdominal obesity and visceral fat-based abdominal obesity according to the different proportion of adipose distribution. Subcutaneous adipose obesity is mainly associated with the gradual accumulation of subcutaneous fat tissue over the abdomen, waist, thigh and buttocks, with a pear-shaped body as a typical representative. Visceral adipose obesity refers to an apple-shaped body with visceral fat tissue accumulation over the internal organs. In contrast to subcutaneous adipose obesity, visceral adipose obesity develops insidiously and causes the patient to be more prone to diabetes, hypertension, arteriosclerosis and ischemic cardio-cerebrovascular disease [10, 11]. Computed tomography (CT) is widely accepted to be a technique to accurately measure subcutaneous and visceral fat and has been used to evaluate fat distribution and quantify adipose tissues in previous studies, as is magnetic resonance imaging (MRI) [12, 13]. However, CT and MRI are expensive and not widely used for large scale epidemiological investigations due to the limitations of the equipment. Body mass index (BMI) as an indicator of obesity is frequently used in current clinical practice, and so waist-to-hip ratio (WHR) does. Neither BMI nor WHR can distinguish visceral adipose obesity from subcutaneous adipose obesity very well. This may lead to underestimate the potential relationship between visceral adipose obesity and disease progression [14–18].

A compound index, the visceral adipose index (VAI), is calculated by the formula composed of triglyceride, BMI, high-density lipoprotein cholesterol and waist circumference (WC), which is strongly associated with abdominal obesity [19]. In identifying visceral fat obesity, VAI is considered to be superior to other indicators, such as BMI and WHR [20]. In view of these advantages, increasing importance has been attached to VAI. A previous study showed that in the general population over 40 years old, VAI was associated with atherosclerotic plaque and carotid stenosis in the cervical artery [16]. The intracranial arteries are the continuation of the carotid arteries into the skull. The anatomical structure and pathophysiological characteristics of the intracranial artery are significantly different from those of the carotid artery. So far, the relationships between aICAS and VAI are still unclear. Thus, this study pursued further exploration of the correlation between aICAS and VAI in the middle of asymptomatic rural residents older than 40 years in China.

## Methods

### Study designs

The Rose asymptomatic IntraCranial Artery Stenosis (RICAS) cohort, a prospective, population-based and ongoing cohort since 2017, is the basis of the present study. All the residents were from Kongcun Town, Pingyin County, China, which is famous as the hometown of rose. The design of RICAS study had described in greater detail before [21].

**Fig. 1:**
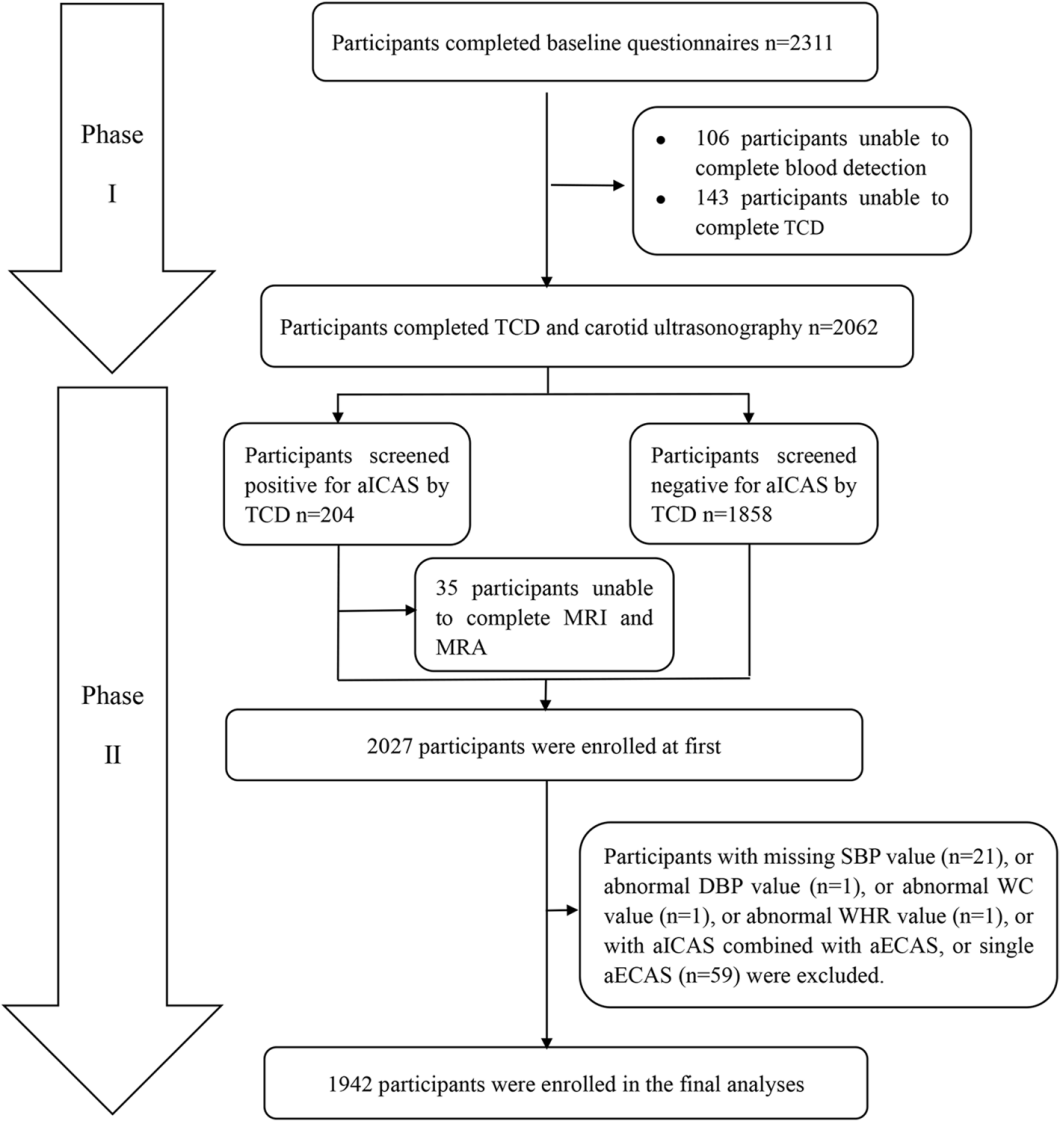
Flow chart of the study participants. MRA, magnetic resonance angiography; TCD, transcranial Doppler; ICAS, intracranial arterial stenosis; aECAS, asymptomatic extracranial arterial stenosis; aICAS, asymptomatic intracranial arterial stenosis; SBP, systolic blood pressure; DBP, diastolic blood pressure; WC, waist circumference; WHR, waist-to-hip ratio

In brief, 2311 rural dwellers from the RICAS study aged ≥ 40 years and without transient ischemic attack and clinical stroke were included. All participants underwent two phases including a demographic characteristic survey and a physical examination including blood examination, transcranial Doppler (TCD) ultrasound and carotid ultrasonography. Baseline information and familiar cardiovascular risk factors (CRFs) were collected incorporating age, sex, HsCRP, diabetes mellitus (DM), smoking habits, hypertension, drinking habits, LDL, BMI, WHR and VAI in the same way. The diagnostic process of asymptomatic extracranial arterial stenosis (aECAS) and aICAS are the same as those in the previous studies [21–23]. In the first stage, aECAS was detected by carotid ultrasonography examination and aICAS was detected by TCD ultrasound. In the second stage, participants diagnosed with aICAS by TCD ultrasound would undergo a further brain magnetic resonance angiography (MRA) examinations and MRI. Overall, 2027 participants completed all the projects, of which those with aECAS (*n* = 59) were then excluded, as well as those with missing diastolic blood pressure (*n* = 1), abnormal WC (*n* = 3), abnormal WHR (*n* = 1) and missing systolic blood pressure (*n* = 21). Ultimately, 1942 participants were recruited for analysis in this current study. The specific flow chart for this study can be located in Fig. 1.

### Assessment of traditional CRFs

In this study, drinking habit was defined as quitting within 6 months or drinking at the very least weekly for 6 months and above. Smoking habit was defined as smoking at the very least one cigarette every day for more than a year or quitting within 6 months. The definition standards of CRFs involving hypertension and DM are the same as those of previous studies [22, 24]. Hypertension was defined as diastolic blood pressure ≥ 90 mmHg, or systolic blood pressure ≥ 140 mmHg, or a self-reported history of hypertension, or having taken any of the antihypertensive drugs. DM was defined as fasting blood glucose ≥ 7.0 mmol/L, or a self-reported history of diabetes mellitus, or a history of taking any type of hypoglycemic medication. BMI was weight divided by height squared. The definition of BMI for weight category adopted the Chinese criterion [25]: normal weight and underweight (BMI ≤ 23.9 kg/m^2^), overweight (24.0 kg/m^2^—27.9 kg/m^2^) and obesity (BMI ≥ 28.0 kg/m^2^). WC and hip circumference (HIP) were also measured and WHR equals WC divided by HIP. Abdominal obesity is defined as participants with WHR > 1, and WHR ≤ 1 was defined as nonabdominal obesity.

### Measurement of VAI

The calculation formula of VAI varied according to gender, as shown below [26]. VAI was further redefined as categorical variable in terms of tertile.

- Males:$$VAI=\frac{WC}{[39.68 + (1.88 \times BMI)]}\times \frac{TG}{1.03}\times \frac{1.31}{HDL}$$
- Females:$$VAI=\frac{WC}{[36.58 + (1.89 \times BMI)]}\times \frac{TG}{0.81}\times \frac{1.52}{HDL}$$

### Assessment of aECAS and aICAS

The aICAS was examined by transcranial doppler (TCD) ultrasound and MRA in different steps, as described above and reference studies [27–29]. Briefly, the participants rested in a quiet room for 5 min, and then two ultrasound experts examined the intracranial arterials through three windows (occipital, eye and temporal windows) using the Visas Companion III TCD system with a 2-MHz probe. In the second phase, follow-up MR and MRA examinations were completed for the participants who had a TCD diagnosis of ≥ 50% stenosis degree in any of the examined vessels according to the criteria [27]. In the examined arteries, the severity of stenosis was ranked into five classes, namely, normal (no stenosis of any degree), mild (stenosis degree < 50%), moderate (50%—70%), severe (stenosis degree ≥ 70%) or occluded [30, 31]. In the present study, the definition of aICAS is the presence of at least one stenosis lesion in any one of the intracranial arteries of any degree on MRA. aECAS was detected by carotid ultrasound and is defined as the presence of a stenotic lesion of any degree in any extracranial arterials [23, 32].

### Data analysis

All the analyses were implemented by the MedCalcProtable V20.0.10 and IBM SPSS Statistics V.25.0 for Windows. For all analyses, statistical differences were regarded as two-tailed *P* value < 0.05. The baseline features are shown as numbers and percentages (%) for categorical variables and medians and IQRs for continuous variables with skewed distributions. Comparisons of baseline characteristics between different groups of participants (with and without aICAS), Mann‒Whitney U test was used for continuous variables, at the same time, the categorical variables are measured by χ^2^ test. In addition, multivariate logistic regression was applied to evaluate the possible association of obese indictors and aICAS. The presence of multicollinearity between covariates was verified before performing multiple logistic regression analysis. The results were obtained from two models: only sex and age were adjusted as confounders in Model 1; hypertension, DM, age, sex, drinking habit, LDL-C, hsCRP, and smoking habit were adjusted for in Model 2. These were all currently recognized risk factors for ICAS, and in the analysis of demographic and clinical characteristics (Table 1), they all showed statistically significant differences in the comparison between groups with and without ICAS. Therefore, we included above as covariates in Model 2. Subgroup analysis was performed under the conditions of Model 2. This study then depicted the receiver operating characteristic (ROC) curves and calculated the area under each curve (AUC) to assess the diagnostic performance of different obesity indictors. There further explored the correlation of VAI with aICAS in the abdominal obesity and nonabdominal obesity subgroups.

**Table 1:** Demographic and clinical characteristics of total participants stratified by aICAS

Characteristics	Total (*n* = 1942)	aICAS		*P* value
		No (*n* = 1810)	Yes (*n* = 132)	
Age (years)	55(49,65)	55(49,65)	60(51,68)	0.001
Sex				0.029
Female, n (%)	1014(52.2)	933(51.5)	81(61.4)	
Male, n (%)	928(47.8)	877(48.5)	51(38.6)	
Hypertension, n (%)	1111(57.2)	1001(55.3)	110(83.3)	< 0.001
DM, n (%)	288(14.8)	249(13.8)	39(29.5)	< 0.001
Dyslipidemia, n (%)	776(40.0)	710(39.2)	66(50.0)	0.015
Smoking habit, n (%)	434(22.3)	419(23.1)	15(11.4)	0.002
Drinking habit, n (%)	641(33.0)	606(33.5)	35(26.5)	0.099
HDL-C (mmol/L)	1.58(1.35,1.87)	1.59(1.36,1.88)	1.45(1.25,1.62)	< 0.001
LDL-C (mmol/L)	2.93(2.52,3.41)	2.93(2.51,3.40)	3.12(2.58,3.68)	0.007
TG (mmol/L)	1.10(0.80,1.61)	1.08(0.80,1.59)	1.35(0.96,2.03)	< 0.001
TC (mmol/L)	5.28(4.67,5.98)	5.29(4.66,5.96)	5.24(4.79,6.13)	0.512
hsCRP (mg/L)	0.64(0.25,1.54)	0.60(0.24,1.51)	0.98(0.45,2.27)	< 0.001
WC (cm)	91(85,98)	91(85,97)	95(90,99)	< 0.001
HIP (cm)	99(95,104)	99(95,104)	101(97,106)	0.001
WHR	0.92(0.87,0.97)	0.92(0.87,0.97)	0.94(0.89,0.98)	0.010
WHR, n (%)				0.102
no-abdominal obesity	1629(83.9)	1525(84.3)	104(78.8)	
abdominal obesity	313(16.1)	285(15.7)	28(21.2)	
BMI (kg/cm^2^)	24.98(22.76,27.34)	24.91(22.68,27.22)	26.14(24.27,28.48)	< 0.001
BMI, n (%)				< 0.001
underweight and normal weight	710(36.6)	680(37.6)	30(22.7)	
Overweight	868(44.7)	804(44.4)	64(48.5)	
Obesity	364(18.7)	326(18.0)	38(28,8)	
VAI	1.18(0.73,1.99)	1.15(0.72,1.93)	1.66(1.04,2.72)	< 0.001
Subgroup of VAI, n (%)				< 0.001
Tertile 1	647(33.3)	625(34.5)	22(16.7)	
Tertile 2	646(33.3)	600(33.1)	46(34.8)	
Tertile 3	649(33.4)	585(32.3)	64(48.5)	

Data was shown as numbers (%) and median (first, third quartiles). Comparison according to aICAS using Mann–whitney *U* test and χ2 test appropriately

*aICAS* Asymptomatic intracranial arterial stenosis, *DM* Diabetes mellitus, *TG* Triglyceride, *TC* Total cholesterol, *HDL-C* High-density lipoprotein cholesterol, *LDL-C* Low-density lipoprotein cholesterol, *WC* Waist circumference, *HIP* Hip circumference, *VAI* Visceral Adiposity Index, *BMI* body mass index, *WHR* Waist-to-hip ratio, *hsCRP* High-sensitivity Creactive protein

## Results

### Demographic characteristics

Of the 1942 participants, 132 were diagnosed with aICAS (6.79%). The average BMI, WHR and VAI for all participants were 24.98 (IQR 22.76–27.34), 0.92 (IQR 0.87–0.97) and 1.18 (IQR 0.73–1.99), respectively. The subjects with aICAS had much higher BMI, WHR and VAI than those without. At the same time, the participants with aICAS appeared to be much older, and their hsCRP level and LDL level were higher than the levels in the patients without aICAS. In addition, the participants with aICAS usually had hypertension, DM, smoking habits, and there was also a higher proportion of female subjects (Table 1).

### Association between obesity indictors and aICAS

In Model 1 and Model 2 of the multivariate adjusted logistic regression, BMI, WHR and VAI were still independently associated with aICAS. Both obesity and overweight were significantly associated with aICAS with a* P* for trend value of 0.010. Similar results were observed for the VAI-Tertile 2 and VAI-Tertile 3 with aICAS (*P* for trend value = 0.005). However, no significant correlation was found between abdominal obesity (WHR > 1) and aICAS (Table 2).

**Table 2:** Association between aICAS and different obesity indices by multivariable logistic-regression model

	Model 1		Model 2	
**Continuous variable**	OR (95%CI)	*P*	OR (95%CI)	*P* value
WHR	1.43(1.12,1.81)	0.004	1.39(1.09,1.78)	0.009
BMI (kg/cm^2^)	1.11(1.06,1.17)	< 0.001	1.06(1.01,1.12)	0.029
VAI	1.22(1.12,1.33)	< 0.001	1.15(1.04,1.26)	0.005
**Categorical variable**	OR (95%CI)	*P*	OR (95%CI)	*P*
**WHR**				
no-abdominal obesity	1(reference)		1(reference)	
abdominal obesity	1.42(0.91,2.20)	0.122	1.42(0.90,2.22)	0.130
**BMI (kg/cm**^**2**^**)**				
underweight and normal weight	1(reference)		1(reference)	
overweight	2.00(1.27,3.14)	0.003	1.60(1.01,2.53)	0.048
obesity	2.90(1.75,4.82)	< 0.001	1.97(1.17,3.32)	0.011
*P* for trend		< 0.001		0.010
Increased per level	1.69(1.32,2.16)		1.40(1.08,1.80)	
**VAI**				
Tertile 1	1(reference)		1(reference)	
Tertile 2	2.09(1.24,3.53)	0.006	1.73(1.01,2.96)	0.047
Tertile 3	3.01(1.83,4.96)	< 0.001	2.15(1.27,3.65)	0.005
*P* for trend		< 0.001		0.005
Increased per tertile	1.68(1.33, 2.12)		1.42(1.11, 1.82)	

Modle1: adjusted for age and sex. Modle2: adjusted for age, sex, hypertension, DM, smoking, alcohol drinking, LDL-C and hsCRP

*OR* Odds ratio, *CI* Confidence interval, *aICAS* Asymptomatic intracranial arterial stenosis, *hsCRP* High-sensitivity C-reactive protein, *DL-C* Low-density lipoprotein cholesterol, *WHR* Waist-to-hip ratio, *BMI* Body mass index, *VAI* Visceral Adiposity Index

### Subgroup analysis of VAI and aICAS

According to Table 3, in the underweight and normal weight subgroup, the VAI-Tertile 3 was independently associated with aICAS. An increase in VAI tertile was positively related to aICAS (*P* for trend = 0.025). Nevertheless, no significant correlations were found in the VAI-Tertile 2.

**Table 3:** Association between VAI and aICAS in subgroups according to different obesity indices by multivariable logistic-regression model

Subgroup	No. of participants	VAI Tertile 1 (*n* = 22)		VAI Tertile 2 (*n* = 46)		VAI Tertile 3 (*n* = 64)		*P* for trend
		OR (95%CI)	*P*	OR (95%CI)	*P*	OR (95%CI)	*P*	
**BMI (kg/cm**^**2**^**)**								
underweight and normal weight	30	1(reference)		2.42(0.89,6.58)	0.084	3.17(1.15,8.71)	0.026	0.025
overweight	64	1(reference)		1.05(0.51,2.19)	0.889	1.12(0.53,2.35)	0.772	0.764
obesity	38	1(reference)		2.23(0.45,10.99)	0.323	2.88(0.62,13.37)	0.176	0.164
**WHR**								
no-abdominal obesity	104	1(reference)		1.73(0.97,3.10)	0.065	2.03(1.14,3.62)	0.017	0.020
abdominal obesity	28	1(reference)		1.48(0.33,6.64)	0.606	2.46(0.57,10.58)	0.225	0.165

Confounding factors: age, sex, hypertension, DM, Smoke, Drink, LDL-C and hsCRP

*OR* Odds ratio, *CI* Confidence interval, *aICAS* Asymptomatic intracranial arterial stenosis, *hsCRP* High-sensitivity C-reactive protein, *LDL-C* Low-density lipoprotein cholesterol, *WHR* Waist-to-hip ratio, *BMI* Body mass index, *VAI* Visceral Adiposity Index

**Fig. 2:**
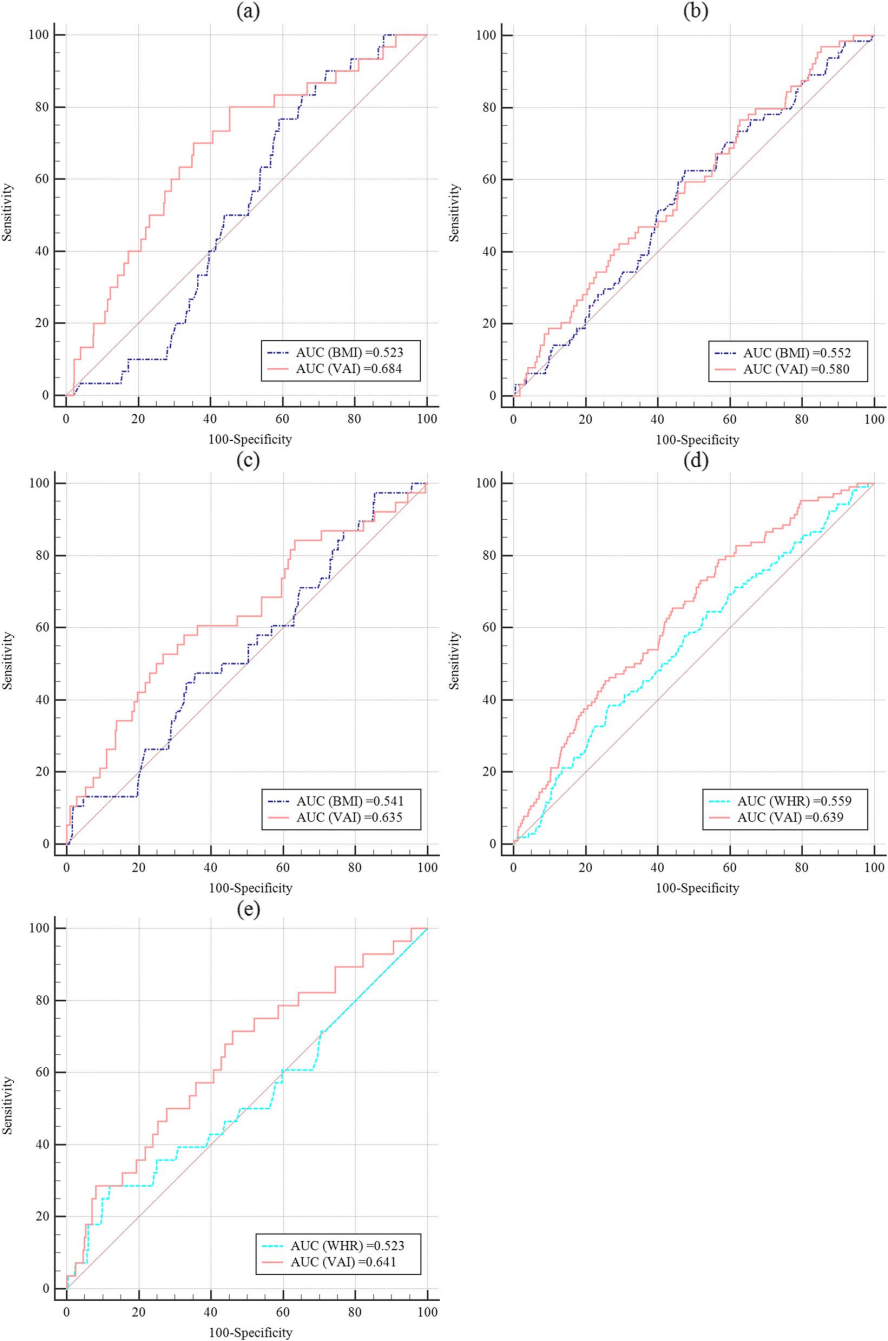
Receiver-operating characteristic (ROC) analysis of the association between VAI and aICAS in comparison to BMI or WHR in the subgroups. **a** The subgroup of underweight and normal weight, *P*_AUC (BMI vs VAI)_ = 0.011; **b** The subgroup of overweight, *P*_AUC (BMI vs VAI)_ > 0.05; **c** The subgroup of obesity, *P*_AUC (BMI vs VAI)_ > 0.05; **d** The subgroup of no-abdominal obesity, *P*_AUC (WHR vs VAI)_ = 0.032; **e** The subgroup of abdominal obesity, *P*_AUC (WHR vs VAI)_ > 0.05; AUC, area under the ROC curve; aICAS, asymptomatic intracranial arterial stenosis; WHR, waist-to-hip ratio; BMI, body mass index; VAI, Visceral Adiposity Index

In Fig. 2, the ROC analysis also showed a relatively good independent diagnostic effect of VAI on aICAS in the BMI subgroups of underweight and normal weight with an AUC of 0.684, which was much better than BMI itself (AUC = 0.523, VAI vs. BMI: *P* = 0.011) (Fig. 2a). Similarly, the correlation of the VAI-Tertile 3 and aICAS was also positive in nonabdominal obesity (WHR ≤ 1) subgroup, with a *P* for trend value of 0.020. The AUC of VAI and aICAS in the nonabdominal obesity group was 0.639, which was also significantly better than that of WHR (VAI vs. WHR: *P* = 0.032). In subgroups of obesity, overweight and abdominal obesity, there were no significant correlations among aICAS with VAI in any tertiles.

## Discussion

This population-based study found a significant positive relationship between VAI and aICAS among asymptomatic residents over 40 years of age in rural China. Of particular interest was that VAI was strongly correlated with aICAS, even when the BMI and WHR levels were within the normal range.

BMI, WHR and VAI are commonly used indicators of obesity that can describe the characteristics of obesity from different points of view. Previous studies have illustrated that BMI, WHR and VAI are significantly associated with CRFs, such as hypertension, DM, and dyslipidemia [33–36]. Both BMI and WHR assess obesity through physical appearance. BMI is the most widely accepted standard to screen for obesity and overweight. A previous study of the RICAS study found a strong linear correlation among BMI with aICAS [21]. Consistent with the previous results, in the present study, obesity and overweight were independently associated with aICAS. WHR mainly reflects abdominal obesity and can also reflect visceral obesity to a certain extent. Although abdominal obesity is considered to be related to hyperlipemia, metabolic syndrome, insulin resistance, diabetes and stroke [37–39]. Few studies have demonstrated that abdominal obese is potentially an element of risk for aICAS. The significant associations between aICAS and abdominal obesity were not found in the present study, although the group of aICAS had significantly higher WHR level.

Different from WHR and BMI, VAI evaluates obesity by a combination of body appearance and metabolic characteristics. It is a relatively specific indicator of visceral fat function and works much better than WHR and BMI. A previous study revealed that the link between VAI and carotid plaque was more pronounced with higher VAI levels in a general population over 40 years old in Northeast China [16]. Furthermore, a study from an asymptomatic Caucasian population investigated the relationship between VAI and coronary artery calcification score and found that VAI may be a beneficial means to evaluate the cardiometabolic risk related to visceral adipose tissue (VAT) [34]. This study found that VAI was an independent factor that positively correlated to the aICAS prevalence, suggesting that in the development of aICAS, visceral obesity rather than subcutaneous fat plays a much more important role. Previous studies discovered that the occurrence and development of aICAS are mainly driven by inflammation, abnormal lipid metabolism, insulin resistance and endothelial dysfunction [40, 41]. VAI can reflect adipose tissue dysfunction [26]. The VAT is an important metabolic organ and an active endocrine gland that can synthesize and secrete large amounts of adipokines with anti-inflammatory and proinflammatory effects [42]. Accumulation of VAT leads to the infiltration of immune cells and an increase in vasoconstrictor secretion, which is more harmful to arteries than the accumulation of SAT [43].

In this study, it was found that with the increase of BMI, the risk of suffering from aICAS disease also increased. Obesity is known as a high-risk factor for cardiovascular disease [19, 40, 44]. Previous review studies have shown that people with a high BMI are at increased risk of various metabolic diseases and increased risk of death from cardiovascular disease compared to those with a low BMI [45, 46]. In a previous study of RICAS cohort, it had shown that BMI and aICAS were positively correlated, which is consistent with the present study [21]. A prospective study in China based on a large coal mining firm found a low prevalence of ICAS in people with a high BMI [47]. The participants in that study were coal miners, generally engaged in heavy physical labour, and it is possible that even though they had a high BMI, they were mostly muscle tissue and not high in body fat. The participants in this study were middle-aged and elderly people in rural areas of China, generally engaged in relatively light physical activities such as farming or housework, and subjects with a high BMI also had a high percentage of fat mass. Another cross-sectional study in patients with at least one vascular risk factor also found that obesity was not associated with middle cerebral artery stenosis [48]. This discrepancy may be due to the presence of other confounding factors included in the high-risk patients, such as occult malignancy, underlying infection or inflammation and harmful lifestyle, which may have an influential role on ICAS over BMI. The participants in this study were an asymptomatic natural population and not all of them had high-risk factors for cerebrovascular disease. In addition, this study also applied MRA to further confirm the presence of aICAS, which is more accurate than TCD.

VAI was significantly correlated with aICAS in the current study when the participants were underweight or normal weight. A related study of the Atahualpa Project showed that in those participants who were not severely obese, BMI was inversely proportional to the severity of carotid siphon calcification [49]. A prospective study of registered female nurses aged 30 to 55 years old in the United States showed that the risk of diabetes increases with an increase in BMI, and even with a normal weight (BMI = 24.0 kg/m^2^) were also at an increased risk [50]. Additionally, a prospective study of Chinese adults who were free of cardiovascular disease (CVD) at baseline divided the participants into four groups based on metabolic and obesity [51]. The study found that in the non-obese population (BMI < 25 kg/m^2^), the change from metabolically healthy to metabolically unhealthy states significantly increased the risk of CVD. Previous studies have suggested an obesity paradox [10], referring to the fact that people with a high BMI show a better prognosis than those of normal weight, but a study found that there is no obesity paradox when obesity takes into account fat distribution [52, 53]. These findings may be due, at least in part, to the fact that BMI exhibits poor diagnostic accuracy in non-obese adults with CVD. In accordance with prior studies, this study also found a higher prevalence of aICAS for participants with the VAI-Tertile 3 even if their BMI was normal or low, which indicated that participants with higher levels of VAI may still have a normal BMI. This confirms the superiority of VIA over BMI in the assessment of visceral fat and, to a certain extent, in the reflection of metabolism. Rapidly changes in the lifestyle and sedentary behavior of Chinese people are likely to lead to rapid abdominal adipose accumulation. At the same time, Asians tend to deposit visceral adipose tissue preferentially when their BMI is low [15, 54]. In this study, in the early stage when BMI was still below the standard of overweight, elevated VAI showed a relatively good diagnostic effect on aICAS with a better AUC. Meanwhile, VAI did not show a better diagnostic role of aICAS in overweight or obese participants. These findings highlighted the warning significance of VAI for aICAS in the pre-obesity stage. Since visceral obesity can be reversed through lifestyle intervention, it is of great necessary to identify high-risk groups in the early stage to prevent obesity-related diseases. VAI can provide more information for the clinical identification of high-risk groups, which may be useful in the early detection and management of aICAS as well as the primary prevention of stroke in community health examinations. This finding may be particularly important for underweight and normal weight people since VAI can provide additional information for the early diagnosis of aICAS in addition to conventional obesity-related indicators such as BMI and WHR. In the future, we would like to combine VAI with radiomics and make full use of machine learning to build a reliable model for predicting aICAS.

### Comparisons with other studies and what does the current work add to the existing knowledge

There are studies which have illustrated that VAI might be a valuable tool in the assessment of visceral fat dysfunction and was associated with metabolic syndrome, diabetes, hypertension, coronary atherosclerosis, kidney disease and so on [12, 16, 26, 33, 34, 55]. The current study found that VAI was correlated to aICAS, even if the participant was normal weight or underweight, which could be a good supplement to BMI.

### Study strengths and limitations

This is the first study on the correlation among VAI with aICAS, filling a prior research gap. The components of VAI are easy to collect in the clinic; therefore, VAI is simple to perform and obtain. In addition, the data of this study is from a large sample cross-sectional study based on a natural population.

Nevertheless, there are still a few limitations. To begin with, it is a single-center study. Multicenter data from different regions and races will be needed in the future. Second, there were relatively few participants with aICAS in total population. Some subgroups had an inadequate sample size and therefore a comprehensive subgroup analysis could not be performed. It is necessary to further expand the sample size in the future to increase the sample size of each subgroup. In addition, the present study did not perform CT or MRI to assess abdominal obesity, which is considered the gold standard. At last, this is a cross-sectional study and therefore no causal inference can be made.

## Conclusion

VAI was positively correlated with the likelihood of having aICAS among rural residents aged over 40 years old in China. Notably, a higher VAI level was still significantly correlated with aICAS among participants who were underweight or normal weight, which indicates that VAI is a considerable indicator for aICAS when participants are still in the pre-obesity stage.

## Data Availability

The data involved in the current study can be obtained with reasonable justification from the corresponding author.

## Abbreviations

MRA: Magnetic resonance angiography
OR: Odds ratio
VAI: Visceral adiposity index
aECAS: Asymptomatic extracranial arterial stenosis
WHR: Waist-to-hip ratio
TCD: Transcranial Doppler
HIP: Hip circumference
aICAS: Asymptomatic intracranial arterial stenosis
CI: Confidence interval
DM: Diabetes mellitus
WC: Waist circumference
LDL-C: Low-density lipoprotein cholesterol
ICAS: Intracranial arterial stenosis
ROC: Receiver operating characteristic
BMI: Body mass index
